# Uso de Estatinas Melhora a Proteção Cardiometabólica Promovida pelo Treinamento Físico em Ambiente Aquático: Um Ensaio Clínico Randomizado

**DOI:** 10.36660/abc.20200197

**Published:** 2021-08-09

**Authors:** Rochelle Rocha Costa, Alexandra Ferreira Vieira, Leandro Coconcelli, Alex de Oliveira Fagundes, Adriana Cristine Koch Buttelli, Laura Frances Pereira, Ricardo Stein, Luiz Fernando Martins Kruel

**Affiliations:** 1 Universidade Federal do Rio Grande do Sul Porto AlegreRS Brasil Universidade Federal do Rio Grande do Sul, Porto Alegre, RS – Brasil.; 2 Hospital de Clínicas de Porto Alegre Porto AlegreRS Brasil Hospital de Clínicas de Porto Alegre, Porto Alegre, RS - Brasil.

**Keywords:** Síndrome Metabólica/complicações, Inibidores de Hidroximethilglutaril-CoA-Redutases, Exercício, Ambiente Aquático, Atividade Física, Hipertensão, Obesidade, Diabetes Mellitus

## Abstract

**Fundamento:**

O uso de estatinas destaca-se como a terapia mais frequentemente utilizada para o tratamento de dislipidemias e pode ser considerado a intervenção farmacológica mais eficiente para a redução da lipoproteína de baixa densidade (LDL). Por outro lado, o treinamento físico pode ser considerado uma estratégia não farmacológica eficiente e segura para promover melhorias no perfil lipídico. No entanto, não se sabe qual seria a influência das estatinas nas adaptações lipídicas decorrentes do treinamento aquático em populações com dislipidemia.

**Objetivos:**

Analisar a influência do uso de sinvastatina nas adaptações lipídicas decorrentes do treinamento aeróbico em meio aquático e de resistência em mulheres idosas com dislipidemia.

**Métodos:**

Sessenta e nove mulheres idosas (66,13 ± 5,13 anos), sedentárias e dislipidêmicas, tanto não usuárias quanto usuárias de sinvastatina (20 mg e 40 mg), foram randomizadas nos 3 grupos seguintes: treinamento aeróbico em meio aquático (WA), treinamento de força em meio aquático (WR) e grupo controle (GC). A duração total das intervenções, para todos os grupos experimentais, foi de 10 semanas, com 2 sessões semanais. As análises bioquímicas foram realizadas antes do início das intervenções e repetidas após o final do ensaio. Foram utilizadas equações de estimativa generalizada para comparar esses dados, estabelecendo α = 0,05.

**Resultados:**

Na análise por intenção de tratar, as participantes medicadas demonstraram uma redução de magnitude maior do colesterol total (CT) (−3,41 a −25,89 mg.dl^−1^; p = 0,038), LDL (−5,58 a −25,18 mg.dl^−1^; p = 0,007) e da relação CT/HDL (−0,37 a −0,61; p = 0,022) quando comparadas às participantes não medicadas, essa redução sendo estatisticamente significativa apenas no grupo WR.

**Conclusões:**

O uso de estatina incrementa as adaptações promovidas pelo treinamento físico aquático no CT, nos níveis de LDL e na relação CT/HDL, sendo mais pronunciado após WR.

## Introdução

As dislipidemias são distúrbios do metabolismo de lipídios, resultando em alterações nas lipoproteínas e lipídeos do sangue.^1^ Em mulheres idosas, os níveis reduzidos de estrogênio, que acompanham a pós-menopausa, podem favorecer o desenvolvimento da dislipidemia e contribuir para o aumento do risco cardiovascular.^2^

A terapia com estatinas é o tratamento mais comumente usado e é considerada a intervenção farmacológica mais eficiente para a redução da lipoproteína de baixa densidade (LDL).^3^^,^^4^ No entanto, vários eventos adversos estão associados ao seu uso, incluindo miopatia, que surge como um efeito colateral preocupante.^5^ Porém, o treinamento físico é considerado uma estratégia não farmacológica eficiente e segura para o tratamento das dislipidemias.^3^ Vários estudos demonstram adaptações favoráveis em lipídios e lipoproteínas em resposta ao treinamento aeróbico^6^^–^^9^ e de resistência.^10^^–^^12^ Contudo, evidências sugerem que as estatinas possam atenuar as melhorias decorrentes do treinamento de exercício em alguns componentes da aptidão física, como o condicionamento cardiorrespiratório^13^ e a força muscular,^14^ embora estes resultados sejam conflitantes.^4^

Está bem documentado que o tratamento isolado com estatinas ou o treinamento físico pode promover melhora do perfil lipídico,^3^ mas existem poucos estudos avaliando os seus efeitos associados. Coen et al.^15^ demonstraram que 10 semanas de treinamento combinado (aeróbico e de resistência) combinado com o uso diário de rosuvastatina não alterou os lipídios, em comparação com o uso de estatina sozinho. É importante ressaltar que o estudo não incluiu um grupo com apenas treinamento físico; portanto, seus efeitos isolados não foram investigados.

Em contraste, Wittke^16^ comparou os efeitos do treinamento aeróbico com ou sem o uso de fluvastatina nos parâmetros lipídicos de homens com dislipidemia. Ambas as estratégias melhoraram os desfechos lipídicos, mas o treinamento aeróbico combinado com o uso da estatina foi mais eficiente na redução das concentrações de colesterol total (CT) e LDL.

Os exercícios aquáticos realizados na posição ortostática estão entre as modalidades de exercícios mais prescritas para idosos.^17^^,^^18^ Adaptações fisiológicas específicas que surgem da imersão levam a um menor impacto articular;^19^ menor pressão arterial;^20^ maior volume sistólico, débito cardíaco e consumo de oxigênio;^20^ supressão do sistema renina-angiotensina;^21^^,^^22^ maior liberação de peptídeo natriurético; e aumento da capacidade oxidativa.^23^^,^^24^ Essas adaptações resultam em benefícios importantes para pacientes idosos e para pacientes com dislipidemias.

É, portanto, relevante conhecer os efeitos do treinamento aeróbico e de resistência em pacientes idosos com dislipidemia. Até onde sabemos, não existem estudos que investiguem a influência da sinvastatina nas adaptações lipídicas promovidas pelo treinamento físico aquático nesta população. Assim, este estudo teve como objetivo analisar a influência do uso de sinvastatina nas adaptações lipídicas decorrentes do treinamento aeróbico em meio aquático e de resistência em mulheres idosas com dislipidemia. A nossa hipótese foi que as participantes que receberam estatinas apresentariam maior magnitude de melhora nas concentrações de CT, triglicerídeos (TG) e LDL do que aquelas que não receberam estatinas.

## Métodos

### Amostra

A amostra foi composta por 69 mulheres idosas (66,13 ± 5,13 anos), sedentárias (sem atividades físicas regulares há pelo menos 3 meses), dislipidêmicas (CT > 200 mg.dl^−1^, LDL ≥ 130 mg.dl^−1^, TG ≥ 150 mg.dl^−1^ ou lipoproteína de alta densidade [HDL] < 40 mg.dl^−1^, isolados ou combinados)^3^ e não tabagistas. Para avaliar a influência do uso de estatinas nas adaptações lipídicas ao treinamento físico, mulheres que não estavam recebendo medicamentos para tratamento de dislipidemias e mulheres que estavam recebendo sinvastatina nas doses de 20 mg e 40 mg foram aceitas para compor o grupo não medicado (NMED) e o grupo medicado (MED), respectivamente. As participantes foram recrutadas em dezembro de 2015 e distribuídas aleatoriamente nos 3 grupos da maneira seguinte: treinamento aeróbico em meio aquático (WA; n = 23; 10 MED e 13 NMED), treinamento de força em meio aquático (WR; n = 23; 9 MED e 14 NMED) e controle (CG; n = 23; 9 MED e 14 NMED). Todas as participantes foram instruídas a não mudar seus hábitos alimentares e a não incluir exercícios adicionais além do prescrito nas intervenções aquáticas.

As participantes foram alocadas nos 3 grupos de estudo por randomização estratificada usando uma lista aleatória gerada por computador. O valor do CT da linha de base foi usado como fator para o processo de randomização. A ocultação da alocação foi realizada por envelopes sequenciais, numerados, opacos e lacrados. Esse procedimento foi realizado por um pesquisador cego, a fim de manter o sigilo da alocação. O processo de randomização e alocação foi realizado após a conclusão das avaliações iniciais.

Este estudo foi realizado de acordo com a Declaração de Helsinque e recebeu aprovação do Comitê de Ética do Hospital de Clínicas de Porto Alegre (protocolo 140547). Todas as participantes leram e assinaram o termo de consentimento livre e esclarecido antes de iniciar a sua participação no estudo. Todas as avaliações e treinamentos foram realizados de dezembro de 2015 a abril de 2016, na Escola de Educação Física, Fisioterapia e Dança da Universidade Federal do Rio Grande do Sul e no Hospital de Clínicas de Porto Alegre. Este ensaio foi registrado na Clinical Trials (protocolo NCT02900612).

### Desenho do Estudo

O presente estudo foi desenhado como um ensaio clínico controlado randomizado de 3 braços em paralelo, com proporção de alocação de 1:1:1. Não foram realizadas mudanças nos métodos após o início do ensaio. Análises bioquímicas foram usadas para medir CT, LDL (desfechos primários), TG, níveis de HDL e a relação CT/HDL (desfechos secundários). Para identificar os hábitos alimentares das participantes, foi adotado um registro alimentar de 3 dias. Essas medidas foram realizadas antes do início das intervenções e repetidas 72 horas após o término do período de 10 semanas. Previamente ao início dos protocolos experimentais, foram realizadas medidas antropométricas para caracterizar a amostra.

### Registro Alimentar

Para garantir que as participantes não alterassem seus hábitos alimentares, foi realizado um registro alimentar de 3 dias diferentes para monitorar os hábitos alimentares. Esse instrumento foi preenchido pelas próprias participantes, e os dados foram calculados por meio do software de nutrição Diet Win Professional (Brubins CAS, Brasil). Os teores de carboidratos (CHO), proteínas (PTN) e lipídios (LIP) foram expressos como porcentagem do valor energético total (VET) diário.

### Avaliações Bioquímicas

Após jejum de 12 horas, 4 ml de sangue foram coletados da veia antecubital. As amostras foram centrifugadas a 1.500 rpm durante 20 minutos e o plasma extraído foi armazenado a −80 °C (ultra freezer NUAIRE, Plymouth, EUA). Um pesquisador cego às condições experimentais realizou a análise do perfil lipídico. Foram analisados CT, TG e HDL pelo método enzimático usando kits da Siemens (Caernarfon, EUA) e analisador químico automático Siemens Advia 1800 (Erlangen, Alemanha). Com base nesses valores, foram estimados os níveis de LDL de acordo com Friedewald et al.^25^ e foi calculada a relação CT/HDL.

### Teste Incremental Aquático

Foi realizado o teste incremental para determinar a frequência cardíaca correspondente ao limiar anaeróbico (FC_LA_), que foi utilizada como indicador da intensidade do treinamento aeróbico, adotando-se o exercício de corrida estacionária. O teste foi realizado antes das sessões de treinamento e repetido na quinta semana de treinamento para a readequação desse parâmetro. O teste incremental já foi descrito em detalhe no estudo de Alberton et al.^19^ A determinação do FC_LA_ foi realizada por 3 fisiologistas do exercício independentes, cegos e experientes. As discordâncias foram resolvidas por consenso.

### Intervenções aquáticas

Antes do início dos treinamentos, os indivíduos que participaram dos grupos WA e WR realizaram 2 sessões de familiarização com os exercícios aquáticos utilizados no programa de treinamento, a fim de garantir a execução adequada dos movimentos. A duração total das intervenções para todos os grupos experimentais foi de 10 semanas, com 2 sessões semanais, resultando em um total de 20 sessões.

O treinamento dos grupos WA e WR foi alterado após 5 semanas com a finalidade de aumentar a intensidade. As sessões de treinamento desses grupos tiveram a mesma estrutura geral, com duração total de 45 minutos, cada uma dividida da forma seguinte: aquecimento (8 minutos), parte principal (aproximadamente 30 minutos) e desaquecimento (7 minutos).

Foi adotado o treinamento intervalado para o grupo WA, com intensidades variando de 90% a 100% da FC_LA_ para o que denominamos o “período de estímulo” e 80% a 90% da FC_LA_ para a recuperação. Foram realizados 6 blocos de 5 minutos, sendo 4 minutos destinados ao estímulo de treinamento e o outro 1 minuto para a recuperação. Durante as primeiras 5 semanas, adotamos 4 minutos em uma intensidade correspondente à FC variando entre 90% e 95% da FC_LA_, intercalados por 1 minuto entre 80% e 85% da FC_LA_; durante as últimas 5 semanas os sujeitos treinaram por 4 minutos entre 95% e 100% do FC_LA_ e por 1 minuto entre 85% e 90% do FC_LA_ durante a recuperação. O controle da intensidade do treinamento do grupo WA foi realizado por meio de monitores de FC (POLAR, FT1, Finlândia).

Durante todo o período de treinamento, o grupo WR realizou os exercícios adotando a velocidade máxima de execução dos movimentos. Também mantiveram um tempo fixo de 1 minuto e 20 segundos para cada exercício. Os intervalos entre as séries foram ativos e realizados em uma intensidade autosselecionada muito leve. Durante as primeiras 5 semanas, foram realizadas 4 séries de 20 segundos, com intervalos de recuperação de 2 minutos e 45 segundos entre as séries. Durante as 5 semanas seguintes, 8 séries de 10 segundos foram realizadas com intervalos de 1 minuto e 40 segundos entre as séries. Os exercícios realizados pelos participantes dos grupos WA e WR já foram descritos detalhadamente por Costa et al.^26^

As participantes do GC realizaram um programa não periodizado de exercícios de relaxamento em imersão, a fim de manter a mesma quantidade de imersão semanal das participantes dos grupos WA e WR, com o objetivo de parear os efeitos fisiológicos da imersão nos desfechos lipídicos dos três grupos experimentais.

### Análise Estatística

O tamanho da amostra foi determinado usando o software GPower (versão 3.1, Universität Düsseldorf, Alemanha) para um poder de cerca 0,80 (nível de significância de 0,05 e coeficiente de correlação de 0,8), com base nos dados da pesquisa de Volaklis, Spassis e Tokmakidis^7^ e Takeshima et al.^17^ Este cálculo mostrou que seriam necessárias 19 mulheres para cada grupo.

Foram adotados os testes de Shapiro-Wilk e de Levene para análise da normalidade e da homogeneidade dos dados, respectivamente. Foram realizados a análise de variância unilateral (para variáveis escalares) e o teste do qui-quadrado (para variáveis categóricas) para comparar os dados dos três grupos (WA, WR e GC) na linha de base (caracterização da amostra). Estes dados foram apresentados como médias e intervalos de confiança de 95%.

Foram usadas equações de estimativa generalizada (GEE) e testes post hoc de Bonferroni para comparar os dados de todas as variáveis dependentes (desfechos primários e secundários) e dos registros alimentares. Assim, os fatores adotados nesta análise foram “grupo” (WA, WR e GC) e “status de medicação” (medicado e não medicado). Estes dados foram apresentados como diferença média (valores pós-intervenção menos pré-intervenção) e intervalos de confiança de 95%, na análise por intenção de tratar. Além disso, foi calculado o tamanho do efeito (d de Cohen) a partir dos valores das diferenças médias entre WA e WR versus GC e classificado como pequeno (entre 0,2 e 0,5), moderado (entre 0,5 e 0,8) ou grande (0,8 ou mais).^27^ Esses resultados foram apresentados em médias e intervalos de confiança de 95%. Para todas as análises, o nível de significância foi estabelecido em α = 0,05 e foi utilizado o software estatístico SPSS (Statistical Package for Social Sciences for Mac, versão 22.0, IBM, EUA).

## Resultados

**Figura 1:**
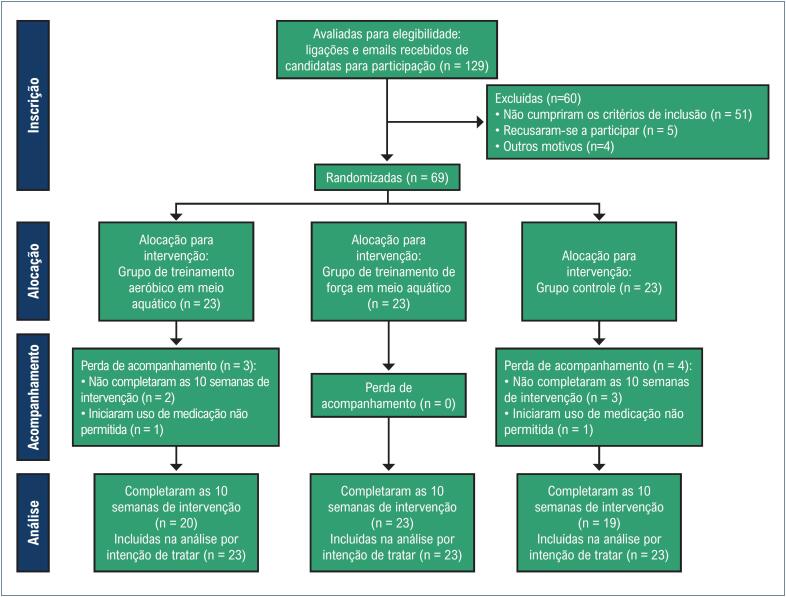
Diagrama de fluxo mostrando o processo de inscrição das participantes, alocação, acompanhamento e análise.

Embora o experimento tenha começado com 69 mulheres aleatoriamente designadas para WA (n = 23), WR (n = 23) e GC (n = 23), 7 participantes retiraram-se do estudo durante o período de intervenção (3 do WA e 4 do GC), representando uma perda de 10%. Assim, 62 participantes concluíram as intervenções do estudo e completaram todas as avaliações (Figura 1). As participantes que completaram a intervenção tiveram frequência de atendimento acima de 95%, demonstrando adesão ao treinamento. As características de linha de base da amostra são apresentadas na Tabela 1.

**Tabela 1:** Características de linha de base dos grupos de treinamento aeróbico em meio aquático (WA), treinamento de força em meio aquático (WR) e de controle (GC)

	WA (n=23) Média ± DP (IC 95%)	WR (n=23) Média ± DP (IC 95%)	GC (n=23) Média ± DP (IC 95%)	valor p
Idade (anos)	66,80 ± 5,51 (64,55 a 69,05)	66,78 ± 5,80 (64,41 a 69,15)	64,63 ± 5,87 (62,23 a 67,03)	0,316
Peso corporal (kg)	71,18 ± 11,40 (66,52 a 75,84)	71,51 ± 15,72 (65,09 a 77,94)	76,91 ± 17,79 (69,64 a 84,18)	0,168
Altura (m)	1,57 ± 0,06 (1,55 a 1,60)	1,55 ± 0,06 (1,52 a 1,57)	1,58 ± 0,07 (1,55 a 1,61)	0,825
IMC (kg.m^−2^)	28,83 ± 4,20 (27,12 a 30,55)	29,88 ± 6,04 (27,41 a 32,35)	30,91 ± 6,95 (28,07 a 33,75)	0,207
Uso de estatina (n/%)	10/43	9/39	9/39	0,639
Uso de estatina 20 mg (n/%)	5/22	4/17	4/17	0,961
Uso de estatina 40 mg (n/%)	5/22	5/22	5/22	0,961

IC: intervalo de confiança; IMC: índice de massa corporal. Foram obtidos os valores de p a partir de análise de variância unilateral (variáveis escalares) e do teste qui-quadrado (variáveis categóricas).

Considerando o registro alimentar, não houve efeitos significativos de grupo (VET p = 0,938; CHO p = 0,872; PTN p = 0,911; LIP p = 0,899) ou tempo (VET p = 0,708; CHO p = 0,790; PTN p = 0,799; LIP p = 0,819) e nenhuma interação significativa entre esses fatores (VET p = 0,803; CHO p = 0,801; PTN p = 0,873; LIP p = 0,858).

**Figura 2:**
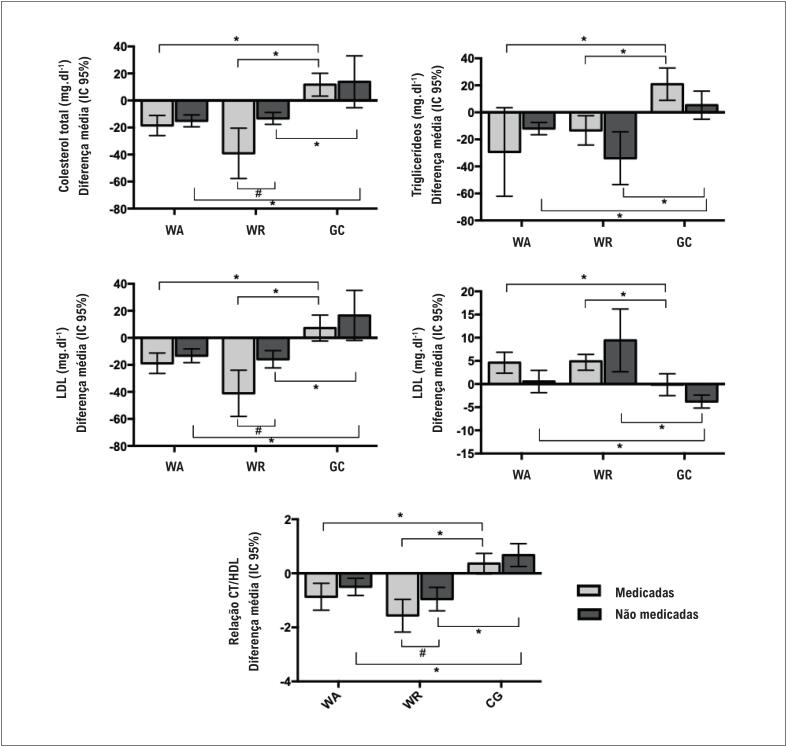
Diferença média (alteração da linha de base) e intervalo de confiança de 95% das concentrações sanguíneas do colesterol total (CT) (A), triglicerídeos (TG) (B), lipoproteína de baixa densidade (LDL) (C), lipoproteína de alta densidade (HDL) (D) e relação CT/HDL (E) de participantes dos grupos de treinamento aeróbico em meio aquático (WA), treinamento de força em meio aquático (WR) e grupo controle (GC), medicadas e não medicados com estatina. * Indica diferença estatisticamente significativa do grupo WR com o mesmo status de medicação. ** Indica diferença estatisticamente significativa dos outros dois grupos com o mesmo status de medicação. # Indica diferença estatisticamente significativa entre o status da medicação dentro do mesmo grupo. As diferenças estatísticas foram obtidas a partir de equações de estimativa generalizadas e testes post hoc de Bonferroni.

Foram encontrados efeitos significativos para o fator de grupo para todos os desfechos analisados no presente estudo (CT: p < 0,001; TG: p < 0,001; LDL: p < 0,001; HDL: p < 0,001; relação CT/HDL: p < 0,001), indicando que WA, WR e GC apresentaram alterações distintas decorrentes dos treinamentos para cada desfecho. O teste de Bonferroni evidenciou comportamento estatisticamente diferente entre os grupos GC e WA e WR, sem diferença entre os grupos com treinamento físico (WA e WR). Para os desfechos de CT, TG, LDL, HDL e relação CT/HDL, o GC apresentou diferenças médias com comportamento oposto ao observado nos outros dois grupos; ou seja, quando os grupos WA e WR mostraram reduções nos resultados, o GC apresentou um aumento (TC, TG, LDL e relação CT/HDL), e quando os grupos WA e WR mostraram aumentos nos resultados, o GC apresentou uma diminuição (HDL) (Figura 2).

Por outro lado, efeitos significativos para o fator de medicação foram encontrados apenas para os desfechos de CT (p = 0,038), LDL (p = 0,007) e relação CT/HDL (p = 0,022). O teste de Bonferroni demonstrou que apenas as participantes do grupo WR apresentaram melhorias de magnitudes diferentes, dependendo do seu status de medicação. As participantes medicadas obtiveram diminuição de maior magnitude em CT, LDL e relação CT/HDL, quando comparadas às não medicadas. Não foram observadas interações significativas entre o grupo e o status de medicação para CT (p = 0,100), TG (p = 0,153), LDL (p = 0,171), HDL (p = 0,083) e relação CT/HDL (p = 0,815) (Figura 2).

A análise do tamanho do efeito, a comparação das participantes de WA e GC, mostrou uma grande magnitude de efeito para todos os desfechos. Da mesma forma, uma grande magnitude de efeito foi observada na comparação das participantes de WR e GC, independentemente do status de medicação (Tabela 2).

**Tabela 2:** Tamanho de efeito do treinamento aeróbico em meio aquático (WA) versus grupo controle (GC) e treinamento de força em meio aquático (WR) versus grupo controle, em participantes medicadas e não medicadas

	Participantes medicadas		Participantes não medicadas	
Desfecho	WA versus GC Tamanho de efeito (IC 95%)	WR versus GC Tamanho de efeito (IC 95%)	WA versus GC amanho de efeito (IC 95%)	WR versus GC Tamanho de efeito (IC 95%)
TC	1,52 (0,87 a 2,18)	1,41 (0,77 a 2,06)	0,83 (0,23 a 1,44)	0,78 (0,18 a 1,38)
TG	0,82 (0,22 a 1,42)	1,20 (0,57 a 1,83)	0,87 (0,27 a 1,47)	1,01 (0,39 a 1,62)
HDL	0,87 (0,27 a 1,47)	0,94 (0,33 a 1,55)	0,89 (0,28 a 1,49)	1,09 (0,47 a 1,71)
LDL	1,22 (0,59 a 1,84)	1,40 (0,76 a 2,04)	0,88 (0,28 a 1,49)	0,94 (0,33 a 1,55)
TC/HDL	1,12 (0,50 a 1,74)	1,53 (0,87 a 2,18)	1,25 (0,62 a 1,88)	1,52 (0,86 a 2,17)

CT: colesterol total; HDL: lipoproteína de alta densidade; LDL: lipoproteína de baixa densidade; TG: triglicerídeos

## Discussão

O achado principal do presente estudo refere-se à influência positiva do uso de estatinas nas adaptações decorrentes da WR, maximizando seus efeitos benéficos nos níveis de CT, LDL e relação CT/HDL. Portanto, foi parcialmente confirmada a hipótese de que as participantes medicadas apresentariam melhorias de magnitudes maiores nos desfechos de CT e LDL, independentemente do modelo de treinamento.

Os efeitos aditivos sobre os benefícios do treinamento físico no CT, na LDL e na relação CT/HDL induzidos pelas estatinas podem ser explicados pelo seu mecanismo de ação. Estes medicamentos são compostos por inibidores da hidroximetilglutaril-coenzima A (HMG CoA) redutase. Esta inibição resulta em redução do colesterol intracelular e, portanto, maior estímulo ao aumento da síntese e da expressão dos receptores de LDL, resultando em aumento da captura do colesterol circulante.^28^

Os efeitos do treinamento físico associado ao uso de estatinas no perfil lipídico foram investigados previamente em populações com dislipidemia. Coen et al.^15^ avaliaram a adição de um programa de treinamento físico combinado ao uso diário de rosuvastatina (10 mg), durante 10 semanas, em indivíduos sedentários de ambos os sexos. O estudo mostrou uma tendência decrescente nos níveis de CT e LDL e um aumento nos níveis de HDL naqueles incluídos no grupo com exercícios mais rosuvastatina. Porém, não é possível comparar os resultados de Coen et al.^15^ com os encontrados no presente estudo, uma vez que não avaliaram um grupo que realizava apenas exercícios. No entanto, Wittke^16^ demonstrou que um programa de exercícios aeróbicos de intensidade moderada, 2 vezes por semana durante 3 meses, foi capaz de promover adaptações positivas nos desfechos do perfil lipídico, principalmente TG (−68,00 mg.dl^−1^) e HDL (+7,70 mg.dl^−1^). Quando esse modelo de treinamento físico foi associado ao uso prévio de fluvastatina (20 mg/dia), efeitos semelhantes foram encontrados no grupo que realizava apenas treinamento físico e no grupo que já usava o medicamento antes do início do protocolo. Por outro lado, foram encontradas alterações com magnitudes pronunciadas em todos os desfechos do perfil lipídico no grupo que iniciou o tratamento farmacológico simultaneamente ao treinamento aeróbico. No entanto, houve diferenças significativas apenas no CT e no LDL em relação ao grupo que realizou o treinamento isolado. Esses resultados corroboram os achados do presente estudo, onde as participantes medicadas iniciaram os protocolos de treinamento já recebendo tratamento com sinvastatina e as que realizaram treinamento aeróbico em meio aquático não apresentaram alterações com magnitudes significativas nas variáveis estudadas, quando comparadas às participantes do grupo que não fazia uso da medicação.

É importante mencionar que nossos achados também demonstram que ambos os modelos de treinamento aquático (WA e WR) promovem melhorias no perfil lipídico de mulheres idosas com dislipidemia, confirmando a nossa hipótese inicial. As melhorias ocorreram de forma semelhante entre as participantes que realizaram o treinamento aeróbico e as que realizaram o treinamento de resistência, demonstrando, assim, a eficácia da prescrição e a periodização dos protocolos propostos.

Os resultados do perfil lipídico dos treinamentos propostos corroboram a literatura que demonstra que os exercícios aquáticos aeróbicos e de resistências são eficientes em promover melhorias nesses parâmetros.^7^^,^^10^^,^^17^^,^^29^^–^^35^ Estudos sugerem que os principais mecanismos explicativos para estes achados estão relacionados à otimização de lipoproteína lipase, proteína de transferência de colesterol éster, lecitina-colesterol aciltransferase, lipase hepática e fosfolipase A2 com treinamento.^36^^–^^37^

Mais especificamente em relação ao meio aquático, a literatura aponta que a simples imersão em posição ortostática promove (ou provoca) a supressão do sistema renina-angiotensina,^21^^,^^22^ o que leva ao aumento do volume sanguíneo e consequente aumento da distensibilidade das câmaras cardíacas.^38^ Isto é um estímulo para a redução dos níveis circulantes de hormônios vasoconstritores, tais como norepinefrina e vasopressina, além da diminuição da atividade da renina plasmática.^39^ Com isso, é sinalizada a necessidade de aumento da secreção e liberação do peptídeo natriurético atrial (ANP), que, de fato, apresenta altas concentrações tanto nas situações de imersão em repouso quanto na realização de exercícios em ambientes aquáticos.^35^^,^^40^^,^^41^ Engeli et al.^24^ afirmam que a ativação da sinalização do ANP contribui para o aumento da capacidade de oxidação de lipídios, influenciando a escolha de substratos para produção de energia durante o exercício. Segundo Moro e Smith,^23^ o ANP é um poderoso regulador do metabolismo lipídico, principalmente na realização de exercícios em imersão. A sua ativação está envolvida em uma cascata de reações enzimáticas da lipase sensível ao hormônio e da lipoproteína lipase, que atuam diretamente na modulação das concentrações de lipídios no sangue. Postula-se que isto possa representar uma via explicativa para os achados benéficos em relação aos protocolos de exercícios aquáticos no perfil lipídico de pacientes com dislipidemia. Porém, embora seu efeito tenha sido relatado na literatura, parece que a imersão isolada (em repouso, sem efeito adicional do exercício) não foi eficiente para promover melhora nos lipídios dos participantes do GC do presente estudo.

O presente estudo tem algumas limitações. Primeiramente, a amostra foi composta exclusivamente por mulheres idosas; portanto, os resultados não podem ser extrapolados para homens ou mulheres mais jovens. Em segundo lugar, não se sabia por quanto tempo a sinvastatina foi usada por toda a amostra antes do início do experimento e não foram testadas outras doses de sinvastatina (10 ou 80 mg). Uma vez que a dosagem do medicamento experimental foi intermediária e sua eficácia demonstrou ser menor em comparação com as estatinas “mais novas”, o efeito de 80 mg de sinvastatina ou a prescrição de outra estatina (atorvastatina, rosuvastatina ou pitavastatina) poderia fornecer um efeito mais positivo sobre o perfil lipídico da amostra.^42^ Finalmente, por motivos financeiros, não foram testadas as concentrações de ANP e a atividade das enzimas de metabolismo lipídico. Estas análises podem fornecer uma visão abrangente dos mecanismos reais pelos quais o perfil lipídico é alterado como resultado dos diferentes modelos de treinamento aquático. Contudo, nosso objetivo não foi o de desenvolver um estudo mecanístico, mas sim o de avaliar a influência da sinvastatina nas adaptações lipídicas decorrentes do treinamento aquático em uma amostra específica com dislipidemia.

## Conclusões

Mulheres idosas dislipidêmicas não medicadas ou intolerantes à sinvastatina podem adotar o treinamento físico aquático como uma ferramenta de tratamento para melhorar o perfil lipídico. Por outro lado, pacientes idosas do sexo feminino com dislipidemia que estão em uso de sinvastatina, mas persistem com níveis não controlados de CT e LDL, também podem se beneficiar dos efeitos do treinamento aeróbico em meio aquático e de resistência, aumentando o efeito hipolipemiante do medicamento.
