# Supplementary material for: Uso de Estatinas Melhora a Proteção Cardiometabólica Promovida pelo Treinamento Físico em Ambiente Aquático: Um Ensaio Clínico Randomizado
Source: Arq Bras Cardiol. 2021 Aug 9;117(2):270–8. [Article in Portuguese] doi: 10.36660/abc.20200197 (PMC8395802; doi:10.36660/abc.20200197)
Supplement: Translation

# Translation: Statin Use Improves Cardiometabolic Protection Promoted By Physical Training in an Aquatic Environment: A Randomized Clinical Trial

**Authors:** Rochelle Rocha Costa, Alexandra Ferreira Vieira, Leandro Coconcelli, Alex de Oliveira Fagundes, Adriana Cristine Koch Buttelli, Laura Frances Pereira, Ricardo Stein, Luiz Fernando Martins Kruel

**Affiliations:** 1 Porto AlegreRS Brazil Universidade Federal do Rio Grande do Sul, Porto Alegre, RS - Brazil.; 2 Porto AlegreRS Brazil Hospital de Clínicas de Porto Alegre, Porto Alegre, RS - Brazil.

**Keywords:** Metabolic Syndrome/complications, Hydroxymethylglutaryl-CoA-Reductases Inhibitors, Exercise, Aquatic Environment, Physical Activity, Hypertension, Obesity, Diabetes Mellitus

## Abstract

**Background:**

Statin use is highlighted as the most commonly utilized therapy for the treatment of dyslipidemias and can be considered as the most efficient pharmacological intervention for low-density lipoprotein (LDL) reduction. On the other hand, physical training can be considered an efficient and safe non-pharmacological strategy to promote improvements in lipid profile. However, the influence of statins on lipid adaptations arising from water-based training in populations with dyslipidemia is not known.

**Objectives:**

To analyze the influence of simvastatin use on lipid adaptations arising from water-based aerobics and resistance training in elderly women with dyslipidemia.

**Methods:**

Sixty-nine elderly (66.13 ± 5.13 years), sedentary, and dyslipidemic women, both non-users and users of simvastatin (20 mg and 40 mg), were randomized into the following 3 groups: water-based aerobic training (WA), water-based resistance training (WR), and control group (CG). Total duration of interventions, for all experimental groups consisted of 10 weeks, with 2 weekly sessions. Biochemical analyses were performed before the beginning of the interventions and repeated after the end of the trial. Generalized estimating equations were used to compare these data, setting α = 0.05.

**Results:**

In intention-to-treat analysis, the medicated participants obtained a greater magnitude of decrease in total cholesterol (TC) (−3.41 to −25.89 mg.dl^−1^; p = 0.038), LDL (−5.58 to −25.18 mg.dl^−1^; p = 0.007) and TC/HDL ratio (−0.37 to −0.61; p = 0.022) when compared to the non-medicated participants, and this decrease was statistically significant only in the WR group.

**Conclusions:**

Statin use enhances the adaptations promoted by water-based physical training in CT, LDL levels, and CT/HDL ratio, and it is more pronounced after WR.

## Introduction

Dyslipidemias are lipid metabolism disorders resulting in altered blood lipoproteins and lipids.^1^ In elderly women, reduced estrogen levels, which accompany post-menopause, can favor the development of dyslipidemia and contribute to increased cardiovascular risk.^2^

Statin therapy is the most commonly used treatment, and it is considered the most efficient pharmacological intervention for low-density lipoprotein (LDL) reduction.^3^^,^^4^ However, several adverse events are associated with its use, including myopathy, which arises as a concerning side-effect.^5^ However, physical training is considered an efficient and safe non-pharmacological strategy for the treatment of dyslipidemias.^3^ Several studies demonstrate favorable adaptations in lipids and lipoproteins in response to aerobic^6^^–^^9^ and resistance training.^10^^–^^12^ Nevertheless, evidence suggests that statins can attenuate the improvements resulting from exercise training in some physical fitness components, such as cardiorespiratory conditioning^13^ and muscle strength,^14^ although these results are conflicting.^4^

It is well documented that isolated treatment with statins or physical training can promote lipid profile improvements,^3^ but there are few studies assessing their associated effects. Coen et al.^15^ demonstrated that 10 weeks of combined training (aerobic and resistance) combined with daily use of rosuvastatin did not alter lipids, compared to statin use alone. It is important to emphasize that this study did not include a group with only physical training; therefore, its isolated effects were not investigated.

In contrast, Wittke^16^ compared the effects of aerobic training with or without fluvastatin use on lipid parameters of men with dyslipidemia. Both strategies improved lipid outcomes, but combined aerobic training and statin use was more efficient in reducing total cholesterol (TC) and LDL concentrations.

Water-based exercises performed in the orthostatic position are among the most prescribed exercise modalities for the elderly.^17^^,^^18^ Specific physiological adaptations which arise from immersion lead to lower joint impact;^19^ lower blood pressure;^20^ greater systolic volume, cardiac output, and oxygen consumption;^20^ suppression of the renin-angiotensin system;^21^^,^^22^ greater release of natriuretic peptide; and increased oxidative capacity.^23^^,^^24^ These adaptations result in important benefits for elderly patients and patients with dyslipidemias.

It is, therefore, relevant to know the effects of aerobic and resistance training in elderly patients with dyslipidemia. To the best of our knowledge, there are no studies investigating the influence of simvastatin on lipid adaptations promoted by water-based physical training in this population. Thus, this study aimed to analyze the influence of simvastatin use on lipid adaptations arising from water-based aerobic and resistance training in elderly women with dyslipidemia. We hypothesized that participants receiving statin would show greater magnitude of improvement in TC, triglycerides (TG), and LDL concentrations than those who did not receive statins.

## Methods

### Sample

The sample comprised 69 elderly (66.13 ± 5.13 years), sedentary (without regular physical activities for at least 3 months), dyslipidemic (TC > 200 mg.dl^−1^, LDL ≥ 130 mg.dl^−1^, TG ≥ 150 mg.dl^−1^, or high-density lipoprotein [HDL] < 40 mg.dl^−1^, isolated or combined),^3^ non-smoking women. In order to assess the influence of statin use on lipid adaptations to physical training, women who were not receiving medication to treat dyslipidemias and women who were receiving simvastatin in dosages of 20 mg and 40 mg were accepted to compose the non-medicated group (NMED) and the medicated group (MED), respectively. Participants were recruited in December 2015, and they were randomly assigned into the following 3 groups: water-based aerobic training (WA; n = 23; 10 MED and 13 NMED), water-based resistance training (WR; n = 23; 9 MED and 14 NMED), and control (CG; n = 23; 9 MED and 14 NMED). All participants were instructed not to change their dietary habits and not to include additional exercise beyond that prescribed in the water-based interventions.

Participants were allocated into the 3 study groups by stratified randomization using a computer-generated random list. The baseline TC value was used as factor for the randomization process. Allocation concealment was performed by sequential, numbered, opaque and sealed envelopes. This procedure was performed by a blinded researcher, in order to maintain the confidentiality of allocation. The process of randomization and allocation were carried out after the completion of initial assessments.

This study was conducted according to the Declaration of Helsinki, and it received approval from the Ethics Committee of the Hospital de Clínicas de Porto Alegre (protocol 140547). All participants read and signed an informed consent form before starting their participation in the study. All the evaluations and the training sessions were performed from December 2015 to April 2016, at the Escola de Educação Física, Fisioterapia e Dança of Universidade Federal do Rio Grande do Sul and at Hospital de Clínicas de Porto Alegre. This trial has been registered at Clinical Trials (protocol NCT02900612.).

### Study Design

This study was designed as a 3-arm randomized controlled clinical trial in parallel, with allocation ratio of 1:1:1. No changes were made to the methods after trial commencement. Biochemical analyses were used to measure TC, LDL (primary outcomes), TG, HDL levels, and the TC/HDL ratio (secondary outcomes). In order to identify the dietary habits of the participants, a 3-day dietary record was adopted. These measurements were performed before the beginning of the interventions and repeated 72 hours after the conclusion of the 10-week period. Prior to the beginning of the experimental protocols, anthropometric measurements were carried out to characterize the sample.

### Dietary Record

To ensure that the participants did not alter their dietary habits, a dietary record of 3 different days was conducted to monitor eating habits. This instrument was completed by the participants themselves, and data were calculated adopting the nutrition software Diet Win Professional (Brubins CAS, Brazil). Carbohydrate (CHO), protein (PTN) and lipid (LIP) content were expressed as percentages of the daily total energy value (TEV).

### Biochemical Assessments

After a 12-hour fasting period, 4 ml of blood were taken from the antecubital vein. Samples were centrifuged at 1,500 rpm for 20 minutes and the extracted plasma was stored at −80 °C (ultra freezer NUAIRE, Plymouth, USA). A researcher who was blinded to the experimental conditions conducted the lipid profile analysis. TC, TG, and HDL were analyzed by enzymatic method using kits from Siemens (Caernarfon, USA) and a Siemens Advia 1800 automated chemistry analyzer (Erlangen, Germany). Based on these values, LDL levels were estimated according to Friedewald et al.,^25^ and the TC/HDL ratio was calculated.

### Aquatic Incremental Test

The incremental test was performed to determine the heart rate corresponding to the anaerobic threshold (HR_AT_), which was used as an indicator of the intensity of aerobic training, adopting the stationary running exercise. The test was performed prior to the training sessions, and it was repeated in the fifth training week in order to readjust this parameter. The incremental test has been described in detail in the study by Alberton et al.^19^ HR_AT_ determination was carried out by 3 independent, blinded, experienced exercise physiologists. Disagreements were decided by consensus.

### Aquatic Interventions

Before training sessions begins, individuals who participated in the WA and WR groups held 2 familiarization sessions with the aquatic exercises utilized in the training program, in order to ensure proper execution of the movements. Total duration of interventions for all experimental groups consisted of 10 weeks, with 2 weekly sessions, resulting in a total of 20 sessions.

Training of WA and WR groups was changed after 5 weeks in order to increase the intensity. The training sessions of these groups comprised the same general structure, with a total duration of 45 minutes, each divided as follows: warm-up (8 minutes), main part (approximately 30 minutes) and cool-down (7 minutes).

Interval training was adopted for WA group, with intensities ranging from 90% to 100% of the HR_AT_ for what we called “stimulus period” and 80% to 90% of the HR_AT_ for recovery. Six blocks of 5 minutes were performed, in which 4 minutes were intended for training stimulus, and the other 1 minute to recovery. In the first 5 weeks, we adopted 4 minutes at an intensity corresponding to HR ranging between 90% and 95% of the HR_AT_, interspersed by 1 minute between 80% and 85% HR_AT_; for the last 5 weeks the subjects trained for 4 minutes between 95% and 100% of the HR_AT_ and for 1 minute between 85% and 90% of the HR_AT_ during recovery. The training intensity control of WA group was conducted using HR monitors (POLAR, FT1, Finland).

During the whole training period, the WR group performed the exercises adopting the maximum execution speed of the movements. They also kept a fixed time of 1 minute and 20 seconds for each exercise. The intervals between the sets were active and performed at a very light self-selected intensity. During the first 5 weeks, 4 sets of 20 seconds were performed, with recovery intervals of 2 minutes and 45 seconds between sets. During the following 5 weeks, 8 sets of 10 seconds were accomplished with intervals of 1 minute and 40 seconds between the sets. The exercises performed by the participants of the WA and WR groups were described in detailed by Costa et al.^26^.

CG participants performed a non-periodized program comprising relaxation exercises in immersion, in order to maintain the same weekly immersion amount of the WA and WR participants, with the aim of matching the physiological effects of immersion on lipid outcomes for the 3 experimental groups.

### Statistical Analysis

The sample size was determined using GPower software (version 3.1, Universität Düsseldorf, Germany) for a power of about 0.80 (significance level of 0.05 and correlation coefficient of 0.8), based on data from research by Volaklis, Spassis, and Tokmakidis ^7^ and Takeshima et al.^17^ This calculation showed that 19 women would be needed in each group.

Shapiro-Wilk and Levene tests were adopted for analysis of the normality and homogeneity of the data, respectively. One-way analysis of variance (for scalar variables) and chi-square test (for categorical variables) were performed to compare data from the 3 groups (WA, WR, and CG) at baseline (sample characterization). These data were shown as means and 95% confidence intervals.

Generalized estimating equations (GEE) and Bonferroni post hoc tests were used to compare the data of all dependent variables (primary and secondary outcomes) and of the dietary recalls. Thus, the factors adopted in this analysis were “group” (WA, WR, and CG) and “medication status” (medicated and non-medicated). These data were presented as mean difference (post-intervention minus pre-intervention values) and 95% confidence intervals, in intention-to-treat analysis. Furthermore, the effect size (Cohen's d) was calculated from mean differences values between WA and WR *versus* CG, and classified as small (between 0.2 and 0.5), moderate (between 0.5 and 0.8), or large (0.8 or more).^27^ These results were shown as means and 95% confidence interval. For all analyses, significance level was set at α = 0.05, and the statistical software SPSS (Statistical Package for Social Sciences for Mac, version 22.0, IBM, USA) was used.

## Results

Although the experiment started with 69 women randomly assigned to WA (n = 23), WR (n = 23), and CG (n = 23), 7 participants withdrew from the study during the intervention period (3 from WA and 4 from CG), representing a dropout of 10%. Thus, 62 participants finished the study interventions and completed all assessments (Figure 1). The participants who completed the intervention had an attendance frequency above 95%, demonstrating adherence to training. Sample baseline characteristics are presented in Table 1.

**Figure 1:** Flow diagram showing the participants enrollment process, allocation, follow-up and analysis.

**Table 1:** Baseline characteristics of the water-based aerobic trainings (WA), water-based resistance traininge (WR) and control (CG) groups

	WA (n=23) Mean ± SD (95% CI)	WR (n=23) Mean ± SD (95% CI)	CG (n=23) Mean ± SD (95% CI)	p value
Age (years)	66.80 ± 5.51 (64.55 to 69.05)	66.78 ± 5.80 (64.41 to 69.15)	64.63 ± 5.87 (62.23 to 67.03)	0.316
Body weight (kg)	71.18 ± 11.40 (66.52 to 75.84)	71.51 ± 15.72 (65.09 to 77.94)	76.91 ± 17.79 (69.64 to 84.18)	0.168
Height (m)	1.57 ± 0.06 (1.55 to 1.60)	1.55 ± 0.06 (1.52 to 1.57)	1.58 ± 0.07 (1.55 to 1.61)	0.825
BMI (kg.m^−2^)	28.83 ± 4.20 (27.12 to 30.55)	29.88 ± 6.04 (27.41 to 32.35)	30.91 ± 6.95 (28.07 to 33.75)	0.207
Statin use (n/%)	10/43	9/39	9/39	0.639
Statin 20 mg use (n/%)	5/22	4/17	4/17	0.961
Statin 40 mg use (n/%)	5/22	5/22	5/22	0.961

BMI: body mass index; CI: Confidence interval. P values were obtained from one-way analysis of variance (scalar variables) and chi-square test (categorical variables).

Considering the dietary record, there were no significant effects of group (TEV p = 0.938; CHO p = 0.872; PTN p = 0.911; LIP p = 0.899) or time (TEV p = 0.708; CHO p = 0.790; PTN p = 0.799; LIP p = 0.819) and no significant interactions between these factors (TEV p = 0.803; CHO p = 0.801; PTN p = 0.873; LIP p = 0.858).

Significant effects were found in factor of group for all outcomes analyzed in the present study (TC: p < 0.001; TG: p < 0.001; LDL: p < 0.001; HDL: p < 0.001; TC/HDL ratio: p < 0.001), indicating that WA, WR, and CG showed distinct alterations resulting from trainings for each outcome. Bonferroni test evidenced a statistically different behavior between the CG and the WA and WR groups, without difference between the groups with physical training (WA and WR). For the outcomes of TC, TG, LDL, HDL, and TC/HDL ratio, the CG presented mean differences with the opposite behavior of those observed in the 2 other groups; that is, when WA and WR groups showed decreases in the outcomes, CG showed an increase (TC, TG, LDL, and TC/HDL ratio), and when WA and WR groups showed increases in the outcomes, CG showed a decrease (HDL) (Figure 2).

**Figure 2:** Mean difference (change from baseline) and 95% confidence interval of blood concentrations of total cholesterol (TC) (A), triglycerides (TG) (B), low-density lipoprotein (LDL) (C), high-density lipoprotein (HDL) (D) and TC/HDL ratio (E) of water-based aerobic training (WA), water-based resistance training (WR) and control group (CG) participants medicated and non-medicated with statin. * Indicates statistically significant difference from the WR group with the same medication status. ** Indicates statistically significant difference from the other 2 groups with same medication status. # Indicates statistically significant difference between medication status within the same group. Statistical differences were obtained from generalized estimating equations and Bonferroni post hoc tests.

On the other hand, significant effects for the factor of medication were only found for the outcomes of TC (p = 0.038), LDL (p = 0.007), and TC/HDL ratio (p = 0.022). The Bonferroni test demonstrated that only WR group participants showed improvements of different magnitudes, depending on their medication status. The medicated participants obtained a decrease of greater magnitude in TC, LDL, and TC/HDL ratio when compared to the non-medicated ones. Significant interactions between group and medication status were not observed for TC (p = 0.100), TG (p = 0.153), LDL (p = 0.171), HDL (p = 0.083) and TC/HDL ratio (p = 0.815) (Figure 2).

The analysis of the effect size, comparison of the participants of WA and CG showed a large magnitude of effect for all the outcomes. Similarly, a large magnitude of effect was observed in the comparison of participants of WR and CG, regardless of medication status (Table 2).

**Table 2:** Effect size of the water-based aerobic training (WA) versus control group (CG) and water-based resistance training (WR) versus control group, in medicated and non-medicated participants

	Medicated participants		Non-medicated participants	
Outcome	WA versus CG Effect size (95% CI)	WR versus CG Effect size (95% CI)	WA versus CG Effect size (95% CI)	WR versus CG Effect size (95% CI)
TC	1.52 (0.87 a 2.18)	1.41 (0.77 a 2.06)	0.83 (0.23 a 1.44)	0.78 (0.18 a 1.38)
TG	0.82 (0.22 a 1.42)	1.20 (0.57 a 1.83)	0.87 (0.27 a 1.47)	1.01 (0.39 a 1.62)
HDL	0.87 (0.27 a 1.47)	0.94 (0.33 a 1.55)	0.89 (0.28 a 1.49)	1.09 (0.47 a 1.71)
LDL	1.22 (0.59 a 1.84)	1.40 (0.76 a 2.04)	0.88 (0.28 a 1.49)	0.94 (0.33 a 1.55)
TC/HDL	1.12 (0.50 a 1.74)	1.53 (0.87 a 2.18)	1.25 (0.62 a 1.88)	1.52 (0.86 a 2.17)

HDL: high-density lipoprotein; LDL: low-density lipoprotein; TC: total cholesterol; TG: triglycerides.

## Discussion

The main finding of the present study refers to the positive influence of statin use on the adaptations arising from WR, maximizing its beneficial effects on TC, LDL, and TC/HDL ratio levels. Therefore, the hypothesis that the medicated participants would show improvements of greater magnitudes in TC and LDL outcomes, regardless of the training model, was partially confirmed.

The additive effects on the benefits of physical training in TC, LDL, and TC/HDL ratio induced by statins can be explained by its mechanism of action. These drugs are composed by hydroxymethylglutaryl-coenzyme A (HMG CoA) reductase inhibitors. This inhibition results in intracellular cholesterol reduction, and, thus, a greater stimulus to the increase of the synthesis and expression of LDL receptors, resulting in increased capture of circulating cholesterol.^28^

The effects of physical training associated to the use of statins on lipid profile were previously investigated in the dyslipidemic population. Coen et al.^15^ evaluated the addition of a combined physical training program to the daily use of rosuvastatin (10 mg), during 10 weeks in sedentary individuals of both sexes. The study showed a decreasing tendency in TC and LDL levels and an increase in HDL levels in those enrolled to exercise plus rosuvastatin. However, it is not possible to compare the results from Coen et al.^15^ with those found in the present study, since they did not evaluate a group that performed only exercise. Nevertheless, Wittke^16^ demonstrated that a moderate intensity aerobic exercise program, twice a week during 3 months, was able to promote positive adaptations in lipid profile outcomes, mainly TG (−68.00 mg.dl^−1^) and HDL (+7.70 mg.dl^−1^). When this model of physical training was associated with the previous use of fluvastatin (20 mg/day), similar effects were found in the group that performed only physical training and the group that already used the medicine prior to the beginning of the protocol. On the other hand, alterations with marked magnitudes in all lipid profile outcomes were found in the group that begun the drug treatment simultaneously to aerobic training. However, there were significant differences only in TC and LDL in relation to the group that performed isolated training. These results corroborate the findings of the present study, where the medicated participants started the training protocols while already receiving treatment with simvastatin, and those who performed water-based aerobic training did not show alterations with significant magnitudes in the studied variables, when compared with the participants from the group that did not use medication.

It is important to mention that our findings also demonstrate that both water-based training models (WA and WR) promote improvements in the lipid profile of elderly women with dyslipidemia, confirming our initial hypothesis. The improvements occurred in a similar manner among the participants that underwent aerobic training and those who performed resistance training, thus showing efficacy of the prescription and periodization of the proposed protocols.

Lipid profile results of the proposed trainings corroborate the literature that demonstrated that water aerobics and water resistance exercises are efficient in promoting improvements in these parameters.^7^^,^^10^^,^^17^^,^^29^^–^^35^ Studies suggest that the main explanatory mechanisms for these findings are related to lipoprotein lipase, cholesteryl ester transfer protein, lecithin cholesterol acyltransferase, hepatic lipase, and phospholipase A2 optimization with training.^36^^–^^37^

More specifically regarding the aquatic environment, the literature points out that simple immersion in orthostatic position promotes (or causes) the suppression of the rennin-angiotensin system,^21^^,^^22^ which leads to an increased blood volume, and consequent increase in the distensibility of cardiac chambers.^38^ This is a stimulus for the reduction in circulating levels of vasoconstrictor hormones, such as norepinephrine and vasopressin, in addition to the decrease of plasmatic renin activity.^39^ Consequently, the need for increased secretion and release of atrial natriuretic peptide (ANP) is signaled, which, in fact, presents high concentrations in both situations of immersion at rest and in the performance of exercises in aquatic environments.^35^^,^^40^^,^^41^ Interestingly, Engeli et al.^24^ claim that the activation of ANP signaling contributes to the increase of lipid oxidative capacity, influencing the choice of substrates for energy production during exercise. According to Moro and Smith,^23^ ANP is a powerful regulator of lipid metabolism, especially in the accomplishment of exercises in immersion. Its activation is involved in a cascade of enzymatic reactions of the hormone-sensitive lipase and lipoprotein lipase, which directly act on the modulation of blood lipid concentrations. It is postulated that this might represent an explanatory route for the beneficial findings in regard to water-based exercise protocols in the lipid profile of patients with dyslipidemia. However, although its effect has been reported in the literature, it seems that immersion alone (at rest, with no additional effect of exercise) was not efficient to promote improvements in the lipids of CG participants of the present study.

This study has some limitations. First, the sample was composed exclusively of elderly women; therefore, the results cannot be extrapolated to men or younger women. Second, it was not known for how long simvastatin was used by the entire sample prior to the experiment entry, and other simvastatin doses (10 or 80 mg) were not tested. Since the dosage of the experimental drug was intermediate and its efficacy has been shown to be lower in comparison to “newer” statins, the effect of 80 mg of simvastatin or the prescription of another statin (atorvastatin, rosuvastatin, or pitavastatin) may provide a more positive effect on the lipid profile of the sample.^42^ Finally, for financial reasons, ANP concentrations and the activity of lipid metabolism enzymes were not tested. These analyses could provide a comprehensive overview of the real mechanisms by which lipid profile is altered, as a result of different water-based training models. However, our goal was not to develop a mechanistic study, but to evaluate the influence of simvastatin in lipid adaptations arising from water-based training in a specific sample with dyslipidemia.

## Conclusions

Non-medicated dyslipidemic or simvastatin-intolerant elderly women can adopt water-based physical training as a treatment tool to improve lipid profile. On the other hand, elderly female patients with dyslipidemia who are on simvastatin, but persist with uncontrolled levels of TC and LDL can also benefit from the effects of water-based aerobic and resistive training, enhancing the drug's lipid-lowering effect.
